# Alcohol use, acculturation and socioeconomic status among Hispanic/Latino men and women: The Hispanic Community Health Study/Study of Latinos

**DOI:** 10.1371/journal.pone.0214906

**Published:** 2019-04-04

**Authors:** Sheila F. Castañeda, Melawhy L. Garcia, Maria Lopez-Gurrola, Mark Stoutenberg, Kristen Emory, Martha L. Daviglus, Robert Kaplan, Aida L. Giachello, Kristine M. Molina, Krista M. Perreira, Marston E. Youngblood, Denise C. Vidot, Gregory A. Talavera

**Affiliations:** 1 Southbay Latino Research Center, School of Public Health, San Diego State University, San Diego, California, United States of America; 2 Department of Health Science, California State University, Long Beach, Long Beach, California, United States of America; 3 Department of Health & Human Performance, University of Tennessee at Chattanooga, Chattanooga, Tennessee, United States of America; 4 School of Public Health, San Diego State University, San Diego, California, United States of America; 5 Institute for Minority Health Research, University of Illinois at Chicago, Chicago, Illinois, United States of America; 6 Einstein College of Medicine, Bronx, New York, United States of America; 7 Public Health Sciences Division, Fred Hutchinson Cancer Research Center, Seattle, Washington, United States of America; 8 Northwestern University, Chicago, Illinois, United States of America; 9 University of North Carolina, Chapel Hill, North Carolina, United States of America; 10 University of Miami, Miami, Florida, United States of America; Universidad Miguel Hernandez de Elche, SPAIN

## Abstract

The objective of this study was to examine the prevalence and patterns of alcohol use among U.S. Hispanic/Latino adults of diverse backgrounds. The population-based Hispanic Community Health Study/ Study of Latinos (HCHS/SOL) enrolled a cohort of Hispanic/Latino adults (*N* = 16,415) ages 18–74 years at time of recruitment, from four US metropolitan areas between 2008–11. Drinking patterns and socio-demographics questionnaires were administered as part of the baseline examination. The relationship between age, sex, socio-demographics, acculturation, current alcohol use, and alcohol risk disorder, defined by the National Institute on Alcohol Abuse and Alcoholism (NIAAA) [no risk (i.e., never drinker), low risk (i.e., women<7 drinks/week; men<14 drinks/week), and at-risk (i.e., women>7 drinks/week; men>14 drinks/week)] were assessed in unadjusted and adjusted multinomial logistic regression analyses. Men reported a higher prevalence than women of at-risk drinking. For women, increased odds of at-risk alcohol use was associated with: a younger age, greater education, full-time employment, and acculturation after adjustment. For men, having a lower income (vs. higher income) or a higher income (vs. not reported) and being employed fulltime (vs. retired) was associated with at-risk alcohol use. For both men and women, there were variations in odds of at-risk drinking across Hispanic/Latino heritage backgrounds, after adjustment. Exact values, odds ratios and p-values are reported within the text. Common factors across sex associated with at-risk drinking included being of Mexican background and being employed full-time. Intervention strategies should consider diversity within the Hispanic/Latino community when designing alcohol abuse prevention programs.

## Introduction

Alcohol use has been associated with a variety of harmful health effects [1–3]. Potential health risks of alcohol use include unintentional injuries, violence against others, risky sexual behaviors, reproductive health issues, and alcohol poisoning [4, 5]. More distal health concerns include cardiovascular, neurological, psychiatric, behavioral, and social problems, as well as cancer and liver disease[5, 6]. Despite these risks and health concerns, evidence suggests that certain levels of alcohol intake may be cardio-protective[7] and reduce the risks of diabetes[8], metabolic syndrome[9] and stroke[10] when consumed in moderation.

Among Hispanics/Latinos, the influence of acculturation to U.S. mainstream society on alcohol use patterns appears to vary by background (heritage), generational status, and gender [11, 12]. Although Hispanic/Latino women have historically shown lower levels of alcohol consumption compared to women of other racial/ethnic heritages^[^^13^^]^, research shows that as Hispanic/Latino women become acculturated to U.S. culture so do their odds of drinking[14, 15]. The influence of acculturation has been less consistent for Hispanic/Latino men, with studies indicating that acculturation may, or may not, be associated with drinking behaviors [15]. According to the review by Zenmore et al[15], the majority of acculturation and alcohol use studies among Hispanics/Latinos were conducted before 2000, with only seven of the 32 studies published on or after the year 2000. The more recent studies focus on small Latino subpopulations such as migrant workers[16] and college students[17]. While the largest study, examining data from Wave II (2004–2005) of the National Epidemiologic Survey of Alcohol and Related Conditions (NESARC), included 6,359 Hispanics, it did not report differences between heritage groups[18].

The relationship between at-risk alcohol use and socio-economic status (SES) is unclear, whereby risk levels may be similar between low and high SES individuals. However, several global studies have shown a clear association between negative alcohol-related health outcomes, such as alcohol-related mortality and socioeconomic deprivation[19–21]. Thus, while at-risk levels may not vary by SES, when considering the negative effects, the relationship with SES is strengthened making low-SES individual much more at risk. One study has shown that low-SES Latinos, specifically of Mexican-origin, in the United States may be at disproportionate risk of harmful drinking patterns pervasive in their country of origin [22]. Given that Hispanics/Latinos are more likely to experience SES disparities in the United States [23], more research is needed.

In addition, little research has been conducted that examines “problem” drinking among Hispanics/Latinos. Problem drinking, when severe enough, is diagnosed as alcohol use disorder, characterized by compulsive alcohol use, loss of control over intake, and negative affect when not using (http://www.niaaa.nih.gov). Studies generally measure “at-risk” or “problem” drinking by frequency of alcohol intake (per day, week, or month). One study has shown that while Latinos, especially men, may have similar rates of “current” drinking profiles as other ethnicities, Latinos have much lower rates of at-risk drinking (e.g., 15 or more per week for men; 8 or more per week for women) as compared to other ethnicities[24]. van Oers et al. (1999) found that lower education was associated with excessive drinking for men, but not for women[25]. Another study found that higher levels of education were associated with a higher likelihood and frequency of drinking for Mexican-American women[26].

Given these mixed findings and the rapid growth and diversity of Hispanics/Latinos in the U.S., more current research is needed to examine the relationship between various social factors and drinking patterns in this population. Hispanics/Latinos include a diverse array of heritage groups, socioeconomic statuses, and degrees of acculturation (i.e., generational status, years living in the US, and language use preferences). Thus, inclusion of these data is essential to better understand Hispanic/Latino population’s risk factors for at-risk alcohol consumption. Our study objective is to fill these important gaps in the available scientific literature by examining the prevalence and patterns of alcohol use in a large, diverse Hispanic/Latino sample residing in the U.S., including the relationship between alcohol use, sex, SES, and proxies of acculturation.

## Materials and methods

The Hispanic Community Health Study/Study of Latinos (HCHS/SOL) is a community-based prospective cohort study of 16,415 self-identified Hispanic/Latino persons, aged 18–74 years at baseline examination (2008 to 2011), from randomly selected households in four U.S. field centers (Chicago, IL; Miami, FL; Bronx, NY; San Diego, CA). The HCHS/SOL cohort includes participants who self-identified as having a Hispanic/Latino background (immigrant, second, or third generation) with the largest heritage groups being Mexican (n = 6,472), Puerto-Rican (n = 2,728), Cuban (n = 2,348), Central American (n = 1,732), Dominican (n = 1,473), and South American (n = 1,072)[27].

### Study population and design

The sample design and cohort selection has been previously described [28]. Briefly, a stratified two-stage area probability sample of household addresses was selected in each of the four field centers. The first sampling stage randomly selected census block groups with stratification based on Hispanic/Latino concentration and proportion of high/low socio-economic status. The second sampling stage randomly selected households, with stratification, from U.S. Postal Service registries that covered the randomly-selected census block groups.

All other reported values (means and prevalence rates) were weighted to account for the disproportionate selection of the sample and to at least partially adjust for any bias effects due to differential nonresponse in the selected sample at the household and person levels. The adjusted weights were also trimmed to limit precision losses due to the variability of the adjusted weights, and calibrated to the 2010 U.S. Census characteristics by age, sex and Hispanic background in each field center’s target population. All analyses also account for cluster sampling and the use of stratified sample selection [28]. Institutional Review Boards at all institutions (i.e., University of North Carolina, University of Miami, Albert Einstein College of Medicine, Northwestern University, and San Diego State University) reviewed and approved the research. Written informed consent was obtained.

### Measures

Socio-demographics and acculturation-related variables were created in HCHS/SOL based on existing major epidemiological studies, such as NHANES, and are publicly available at: https://www2.cscc.unc.edu/hchs/manuals-pub. The following characteristics were measured via self-report during the baseline examination: date of birth, sex, Hispanic/Latino background group (Cuban, Central American, Dominican, Mexican, Puerto Rican, South American, and other), marital status and socioeconomic indicators (income, education, and employment status). In addition, acculturation proxy measures administered as part of the baseline exam included: Place of birth [defined as: US born (including US territories), non US-born and in the US ≥ 10 years, or non US-born and in the US < 10 years], and primary language of preference for survey administration (English and Spanish).

A questionnaire was administered to determine alcohol use. If participants reported current alcohol use (“*Do you presently drink alcoholic beverages*?*”*) they were asked to provide the amount of glasses, bottles, cans or drinks of: 1) red wine, 2) white wine, 3) beer, and 4) liquor, spirits, or mixed drinks they consumed in a week. For each type of drink assessed, responses included: 1 = every day, 2 = 5–6 days a week, 3 = 3 to 4 days a week, 4 = 2 days a week, 5 = 1 day a week, 6 = 2 to 3 days a month, 7 = 1 day a month, 8 = less than once a month, or 9 = never.

Alcohol use was categorized in two ways for analyses. First, a three-category variable of: never (never drank alcohol), former (not presently drinking alcohol but used to drink alcohol in the past), and current (currently drinking alcohol) was created. Second, an alcohol risk variable, measuring the risk level for developing an alcohol use disorder, was created on a gender-specific basis with three levels: no risk = never used alcohol, low risk = current use < 7 drinks/week (women), < 14 drinks /week (men), and at-risk = ≥ 7 drinks/week (women), ≥14 drinks /week (men) (https://www.niaaa.nih.gov/alcohol-health/overview-alcohol-consumption/moderate-binge-drinking).

### Statistical analyses

Reported values were weighted to adjust for sampling probability and item non-response [27, 28]. Descriptive characteristics, age-standardized to the 2010 U.S. Census population, were computed for the overall population and presented stratified by sex (Table 1). Sex-specific multinomial logistic regression analyses were used to examine associations of alcohol use and risk for alcohol use disorder with age, Hispanic/Latino heritage group, income, education, employment, acculturation [language preference; and nativity]. Adjusted odds ratios (AORs) with 95% confidence intervals (CIs) were computed by sex (Tables 2 and 3). All analyses were performed using SAS version 9.3[29]. All HCHS/SOL participants with incomplete data on key variables (n = 404) were excluded from analyses. For odds ratios less than 1.0 we calculated and reported the inverse for ease of interpretation of the results.

**Table 1:** Sample socio-demographic characteristics, overall and by sex (n = 16,011).

	Overall (N = 16,011)	Women (n = 9,590)	Men (n = 6,421)
	% (SE)	% (SE)	% (SE)
**Age**			
**18–44**	59.7 (0.8)	57.8 (0.9)	61.7 (0.9)
**45+**	40.3 (0.8)	42.2 (0.9)	38.3 (0.9)
**Background**			
**Dominican**	9.5 (0.7)	11 (0.8)	7.9 (0.7)
**Central American**	7.5 (0.6)	7.5 (0.6)	7.4 (0.6)
**Cuban**	20.3 (1.7)	18.5 (1.6)	22.2 (1.9)
**Mexican**	37.8 (1.7)	38.7 (1.7)	36.8 (1.8)
**Puerto-Rican**	15.9 (0.8)	15.1 (0.8)	16.7 (1)
**South American**	4.9 (0.3)	5.2 (0.4)	4.7 (0.4)
**Other**	4.2 (0.3)	4 (0.4)	4.3 (0.4)
**Nativity**			
**Foreign <10 yr**	27.8 (1)	28.4 (1.1)	27 (1.1)
**Foreign > = 10 yr**	49.4 (0.8)	50.8 (0.9)	48 (1)
**US born**^**a**^	22.8 (0.8)	20.8 (0.8)	25 (1.1)
**Language preference**			
**Spanish**	74.9 (0.9)	76.8 (1)	72.9 (1.2)
**English**	25.1 (0.9)	23.2 (1)	27.1 (1.2)
**Income**			
**<30,000**	61.1 (1)	65 (0.9)	56.8 (1.3)
**> = 30,000**	32.7 (1)	27.8 (1)	38.1 (1.3)
**Unknown**	6.2 (0.4)	7.2 (0.4)	5 (0.4)
**Education**			
**<High school**	32.2 (0.7)	32.7 (0.9)	31.7 (0.9)
**High school**	28.1 (0.6)	26.3 (0.7)	30 (0.8)
**> High school**	39.7 (0.8)	41 (1)	38.2 (1)
**Employment status**			
**Retired**	8.3 (0.4)	8.4 (0.5)	8.2 (0.5)
**Unemployed**	40.9 (0.7)	48.4 (0.9)	32.8 (1)
**Part-time**	17.1 (0.4)	18.9 (0.6)	15.1 (0.6)
**Full time**	33.7 (0.7)	24.3 (0.7)	43.9 (1)
**Marital status**			
**Single**	34.4 (0.7)	31.3 (0.8)	37.7 (1)
**Married, living with partner**	49 (0.8)	47.2 (0.9)	50.9 (1.1)
**Separated, divorced, or widowed**	16.7 (0.5)	21.5 (0.7)	11.4 (0.6)
**Alcohol Use**			
**Never**	18.4 (0.7)	26.1 (1)	10.1 (0.6)
**Former**	29.9 (0.7)	32.9 (1)	26.7 (0.8)
**Current**	51.7 (0.8)	41.1 (1.1)	63.2 (1)
**Alcohol Use Disorder Risk**			
**No risk**	26.3 (1)	38.8 (1.4)	13.8 (0.9)
**Low risk**	64.9 (1)	55.8 (1.3)	74 (1)
**At-risk**	8.8 (0.4)	5.4 (0.4)	12.2 (0.7)

Note: Risk for developing alcohol disorder is defined as: No risk = never used alcohol, Low risk = <7 drinks/week (women), < 14 drinks/week (men); and At-risk = 7+drinks/week (women), 14+drinks/week (men). All values are weighted for study design and nonresponse and calibrated using the 2010 U.S. Census population.

^a^US-born includes 50 states or US territories.

**Table 2:** Multinomial logistic regression assessing the relationship between alcohol use and related risk behavior and socio-demographic variables and acculturation for women (n = 9,590).

	Alcohol Use		Alcohol Use Disorder Risk	
	Former OR (95% CI)	Current OR (95% CI)	Low Risk OR (95% CI)	At-Risk OR (95% CI)
**Age**				
**18 -44**	Ref (-)	Ref (-)	Ref (-)	Ref (-)
**45+**	0.73 (0.62–0.87) ***	0.65 (0.55–0.76) ***	0.69 (0.58–0.82) ***	0.36 (0.25–0.52) ***
**Background**				
**Dominican**	1.52 (1.13–2.04) ***	1.52 (1.12–2.05) ***	1.4 (1.04–1.9) ***	3.41 (1.97–5.9) ***
**Central American**	0.38 (0.29–0.49) ***	0.40 (0.32–0.51) ***	0.38 (0.3–0.48) ***	0.64 (0.37–1.09) *
**Cuban**	0.26 (0.2–0.34) ***	0.41 (0.32–0.52) ***	0.4 (0.31–0.52) ***	0.43 (0.25–0.76) ***
**Mexican**	Ref (-)	Ref (-)	Ref (-)	Ref (-)
**Puerto-Rican**	1.11 (0.82–1.51) ***	0.97 (0.71–1.33)	0.91 (0.66–1.27)	1.22 (0.66–2.26
**South American**	0.57 (0.4–0.8)	0.71 (0.51–0.99)	0.68 (0.49–0.95)	0.91 (0.45–1.82)
**Other**	0.55 (0.27–1.1)	0.78 (0.43–1.39)	0.74 (0.41–1.36)	0.93 (0.36–2.43)
**Nativity**				
**<10 yr**	0.65 (0.44–0.96) **	0.46 (0.32–0.66) **	0.48 (0.33–0.7) ***	0.23 (0.12–0.45) ***
**≥10 yr**	0.85 (0.58–1.25)	0.51 (0.36–0.73) *	0.53 (0.37–0.75) *	0.41 (0.23–0.72)
**US born**	Ref (-)	Ref (-)	Ref (-)	Ref (-)
**Lang preference**				
**Spanish**	0.94 (0.63–1.39)	0.66 (0.45–0.96) *	0.66 (0.45–0.97) *	0.82 (0.49–1.38)
**English**	Ref (-)	Ref (-)	Ref (-)	Ref (-)
**Income**				
**<30,000**	0.91 (0.73–1.14)	0.71 (0.57–0.88)	0.74 (0.59–0.94)	0.68 (0.45–1.03)
**≥30,000**	Ref (-)	Ref (-)	Ref (-)	Ref (-)
**Unknown**	0.89 (0.63–1.25)	0.69 (0.49–0.96)	0.72 (0.5–1.02)	0.64 (0.28–1.46)
**Education**				
**<High school**	0.97 (0.79–1.18)	0.7 (0.56–0.86) *	0.68 (0.54–0.85) **	0.66 (0.44–0.99)
**High school**	1.09 (0.88–1.36)	0.73 (0.59–0.9)	0.77 (0.61–0.96)	0.62 (0.38–0.99)
**> High school**	Ref (-)	Ref (-)	Ref (-)	Ref (-)
**Employment**				
**Retired**	0.83 (0.59–1.18)	0.52 (0.37–0.72) ***	0.54 (0.38–0.75) ***	0.33 (0.13–0.83) *
**Unemployed**	0.84 (0.69–1.02)	0.66 (0.54–0.81) *	0.67 (0.54–0.82) *	0.69 (0.44–1.07)
**Part-time**	0.89 (0.7–1.12)	0.95 (0.76–1.2) **	0.98 (0.77–1.25) ***	0.95 (0.56–1.61)
**Full-time**	Ref (-)	Ref (-)	Ref (-)	Ref (-)

Note. Multinomial logistic regression models were used to assess odds of alcohol use (former and current versus never (reference)) and alcohol use disorder risk (low and at-risk versus no risk (reference)).

* p≤ .05

** p≤ .01

*** p≤ .001

**Table 3:** Multinomial Logistic regression assessing the relationship between alcohol use and related risk behavior and socio-demographic variables and acculturation for men (n = 6,421).

	Alcohol Use		Alcohol Use Disorder Risk	
	Former OR (95% CI)	Current OR (95% CI)	Low Risk OR (95% CI)	At-Risk OR (95% CI)
**Age**				
**18 -44**	Ref (-)	Ref (-)	Ref (-)	Ref (-)
**45+**	1.74 (1.3–2.32) ***	1.05 (0.81–1.37)	1.07 (0.81–1.4)	1.01 (0.71–1.43)
**Background**				
**Dominican**	1.03 (0.54–1.98) *	1.38 (0.72–2.65) ***	1.47 (0.77–2.8) ***	0.88 (0.39–1.98)
**Central American**	0.29 (0.18–0.46) ***	0.27 (0.17–0.41) ***	0.27 (0.18–0.43) ***	0.24 (0.14–0.41) ***
**Cuban**	0.12 (0.08–0.18) ***	0.22 (0.15–0.32) ***	0.21 (0.15–0.31) ***	0.29 (0.18–0.46) ***
**Mexican**	Ref (-)	Ref (-)	Ref (-)	Ref (-)
**Puerto-Rican**	0.96 (0.58–1.58) ***	0.63 (0.39–1.02)	0.63 (0.39–1.04)	0.72 (0.38–1.38)
**South American**	0.62 (0.35–1.1)	0.63 (0.36–1.1)	0.66 (0.38–1.16)	0.28 (0.12–0.65)
**Other**	0.92 (0.41–2.06)	0.89 (0.42–1.88)	0.86 (0.4–1.87)	1.18 (0.48–2.89)
**Nativity**				
**<10 yr**	1.9 (1.16–3.1) *	1.27 (0.82–1.98)	1.34 (0.85–2.11)	1.02 (0.55–1.9)
**≥10 yr**	1.55 (1.01–2.37)	1.13 (0.76–1.7)	1.17 (0.77–1.77)	1.05 (0.62–1.79)
**US born**	Ref (-)	Ref (-)	Ref (-)	Ref (-)
**Lang preference**				
**Spanish**	0.85 (0.55–1.31)	0.94 (0.63–1.4)	0.96 (0.65–1.43)	0.84 (0.5–1.41)
**English**	Ref (-)	Ref (-)	Ref (-)	Ref (-)
**Income**				
**<30,000**	1.21 (0.88–1.65) ***	0.84 (0.65–1.1)	0.8 (0.61–1.04)	1.21 (0.84–1.76) ***
**≥30,000**	Ref (-)	Ref (-)	Ref (-)	Ref (-)
**Unknown**	0.62 (0.38–1.01) **	0.48 (0.3–0.74) ***	0.52 (0.33–0.83) **	0.33 (0.15–0.73) ***
**Education**				
**<High school**	1 (0.72–1.38)	0.91 (0.67–1.22)	0.86 (0.63–1.17)	1.13 (0.76–1.67)
**High school**	0.69 (0.51–0.95) **	0.69 (0.53–0.91) **	0.67 (0.51–0.88) *	0.83 (0.58–1.18)
**> High school**	Ref (-)	Ref (-)	Ref (-)	Ref (-)
**Employment**				
**Retired**	0.87 (0.56–1.34)	0.59 (0.39–0.9) *	0.63 (0.41–0.98)	0.38 (0.21–0.68) ***
**Unemployed**	0.97 (0.7–1.35)	0.84 (0.63–1.11)	0.81 (0.61–1.08)	0.93 (0.63–1.36)
**Part-time**	0.91 (0.6–1.36)	0.93 (0.64–1.35)	0.95 (0.65–1.38)	0.79 (0.49–1.28)
**Full-time**	Ref (-)	Ref (-)	Ref (-)	Ref (-)

Note. Multinomial logistic regression models were used to assess odds of alcohol use (former and current versus never (reference)) and alcohol use disorder risk (low and at-risk versus no risk (reference)).

* p ≤ .05

** p≤ .01

*** p≤ .001

## Results

### Sample characteristics

Table 1 presents the weighted descriptive characteristics, stratified by sex. Over half (58%) were women, 40% had greater than a high school education, 23% were U.S. born (including mainland and U.S. territories), 25% preferred English as their first language, 50% were working either full or part-time, and 49% were married or living with a partner. Almost 30% were former drinkers (33%_women_, 27%_men_), and 52% were current drinkers (41%_women_, 63%_men_). Overall, 26% had no risk for alcohol use disorder (AUD) (39%_women_, 14%_men_), 65% were low risk for AUD (56%_women_, 74%_men_), and 9% were at-risk for AUD (5%_women_, 12.2%_men_).

### Correlates of alcohol use and risk for alcohol use disorder (AUD)

#### Women

**Current drinking.** In adjusted multinomial logistic regression analyses among women, being 45+ years old (OR = .65) as compared to 18–44 and being Central American (OR = .40) or Cuban (OR = .41) as compared to Mexican background, and being foreign born in the US < 10 years (OR = .46) or > = 10 years (OR = .51) as compared to US-born were less likely to be current drinkers. Similarly, being Spanish speaking (OR = .66) versus English speaking, having a high school education or less (OR = .7) as compared to greater than high school, and being retired (OR = .52), unemployed (OR = .66), or working part-time (OR = .95) as compared to working fulltime were less likely to be currently drinking alcohol as compared to never drinking. Being of Dominican background (OR = 1.52) as compared to Mexican background was associated with greater odds of current alcohol use. All differences reported were statistically significant at p < .05 or below (see Table 2).

**At-risk drinking.** In adjusted multinomial logistic regression analyses, among women, being over the age of 45 was associated with less odds of at risk drinking (OR = .36) as compared to those aged 18–44 years. Being of Central American (OR = .64) or Cuban background (OR = .43) as compared to Mexican background, being foreign born and in the US less than 10 years as compared to US born (OR = .23) and being retired as compared to working fulltime (OR = .33) was associated with a lower likelihood of at risk drinking (versus no risk). Being Dominican background (OR = 3.41) as compared to Mexican background was associated with greater odds of at-risk drinking (p < .05) (Table 2).

#### Men

**Current drinking.** In adjusted multinomial analyses among men, being of Central American (OR = .27) or Cuban (OR = .22) was compared to Mexican background was associated with less likelihood of current alcohol use. Similarly, having a non-reported income (OR = .48) versus a greater income (of ≥ 30,000 per year), having a high school degree (OR = .69) as compared to greater than high school education (or GED), and being retired (OR = .59) versus employed fulltime was associated with less likelihood of current drinking (versus never drinking). Being of Dominican background (OR = 1.38) as compared to Mexican background was associated with a greater likelihood of current alcohol use (p < .05) (see Table 3).

**At-risk drinking.** For men, in adjusted analyses, being Central American (OR = .24) or Cuban (OR = .29) as compared to being of Mexican background was associated with a lower likelihood of at-risk drinking (compared to no risk). Similarly, having a non-reported income (OR = .33) versus a greater income (of ≥ $30,000 per year) and being retired (OR = .38) versus employed fulltime was associated with less likelihood of at-risk drinking (versus no risk). Having a lower income (<$30,000/year) (OR = 1.21) versus higher income ($≥30,000/year) increased the odds of at-risk alcohol use (versus no risk) (p < .05) (Table 3).

## Discussion

Data from the HCHS/SOL reveals new insights into the patterns and prevalence of alcohol use among diverse Hispanic/Latino heritage groups in the context of sex, socio-demographics and acculturation. Our results imply that for women, a younger age, greater acculturation, having greater than high school education, and being employed fulltime predicted current drinking (vs. never drinking) and at-risk drinking (vs. no risk) (Table 2). For men, age and acculturation were not associated with current drinking or at-risk drinking; however, socioeconomic indicators differentially predicted current or at-risk drinking. For men, being employed full time (versus retired) and having a lower income predicted at-risk use. Having a higher education (greater than high school versus high school/GED) predicted current use (Table 3).

Our study provides a significant contribution to the literature in acknowledging the important cultural differences in alcohol related behaviors across Hispanic/Latino ethnic groups. Our findings are consistent with research that shows differences in patterns of alcohol use by ethnicity, as well differences in predictors of alcohol use among Latino men and women[24, 30, 31]. Specifically, this study adds to the literature supporting that Latino men consume alcohol more regularly than women across all Latino background groups,[24, 30] with greater differences seen among Mexican men. In addition, this study adds to the literature by showing that for men and women, Mexican heritage as compared to Central American or Cuban heritage, was associated with current drinking and at-risk drinking. More research is needed to determine why these between ethnic group differences in risk behaviors exist.

The relationship between acculturation and alcohol use among Hispanics/Latinos has been examined extensively [11, 15]. In this study, women with greater acculturation were more likely to be current drinkers and at higher risk for alcohol use disorders. These results are consistent with findings from a comprehensive review of 32 studies focused on acculturation and alcohol use among Latinos [15]. In this study Zemore (2007) reports that among all studies reviewed, there was a consistent association between higher acculturation and higher odds of drinking among women. Future research in this sample is needed to examine the mechanism by which acculturation leads to alcohol consumption among diverse Latinas in the U.S. One plausible explanation is the relationship of acculturative stress and alcohol use examined in other studies [32].

In this study, HCHS/SOL male participants that were employed full time, and were of low-income were more likely to be at risk for alcohol use disorder. One possible explanation is that there is a compounding effect of demanding, yet low-paying full-time jobs that leads to at-risk alcohol consumption. Similar results have been reported in previous studies [31, 33] that examined the effects of poverty on at-risk alcohol consumption. A review article by Collins (2016) focused on the relationship between SES (i.e., income, education, and employment), alcohol use, and related health outcomes highlights the complex set of systems that determine alcohol use among different racial/ethnic groups. The overall consensus is that lower SES, and associated living conditions (e.g., more debt, stress, neighborhood deprivation) are factors associated with increased risk of drinking[34]. For example a study using data from the 2005 U.S. National Alcohol Survey found that Hispanics exposed to social disadvantage are at greater risk for problem drinking[35]. Future research should examine the interplay of individual and community level factors on alcohol use and related behaviors.

One limitation of this study is the cross-sectional design which does not allow for the exploration of the directionality of the associations among SES, acculturation, gender, and alcohol use. Additionally, this study is limited to four major metropolitan cities in the United States and does not include rural Hispanics/Latinos. One of the strengths of the current study is the probability-based sampling which allows for the estimation of prevalence in the target population in the four communities (Bronx, Chicago, Miami, and San Diego). These four communities are diverse and provide adequate representation for comparing the different Hispanic/Latino heritage groups.

## Conclusions

To our knowledge, the HCHS/SOL study is the largest contemporary study to examine alcohol use and contributing factors among diverse Hispanic/Latino heritage groups. Results from this study show that prevalence and patterns of alcohol use vary among Hispanics/Latinos of diverse heritage, as well as by sex. Given the growing numbers of Mexican background individuals in the US, more research is needed to further examine factors that may contribute to at-risk alcohol use among this group. Further, more research is needed to examine acculturation levels and potential mediators (e.g., acculturative stress) and at-risk drinking for Hispanic/Latina women. Overall, these findings underlie the importance of tailoring research and intervention programs to examine socio-economic and sex-specific factors contributing to alcohol use among Hispanics/Latinos.

## References

[1] Ikehara S, Iso H, Yamagishi K, Kokubo Y, Saito I, Yatsuya H, et al Alcohol consumption and risk of stroke and coronary heart disease among Japanese women: the Japan Public Health Center-based prospective study. Preventive medicine. 2013;57(5):505–10. Epub 2013/07/19. 10.1016/j.ypmed.2013.07.003 . 23859928

[2] Mukamal KJ, Chung H, Jenny NS, Kuller LH, Longstreth WT Jr., Mittleman MA, et al Alcohol consumption and risk of coronary heart disease in older adults: the Cardiovascular Health Study. Journal of the American Geriatrics Society. 2006;54(1):30–7. Epub 2006/01/20. 10.1111/j.1532-5415.2005.00561.x . 16420195

[3] Rehm J, Gmel GE Sr., Gmel G, Hasan OSM, Imtiaz S, Popova S, et al The relationship between different dimensions of alcohol use and the burden of disease-an update. Addiction. 2017;112(6):968–1001. 10.1111/add.13757 28220587 PMC5434904

[4] Scott-Sheldon LA, Carey KB, Cunningham K, Johnson BT, Carey MP, Team MR. Alcohol Use Predicts Sexual Decision-Making: A Systematic Review and Meta-Analysis of the Experimental Literature. AIDS Behav. 2016;20 Suppl 1:S19–39. 10.1007/s10461-015-1108-9 26080689 PMC4683116

[5] Center for Disease Control and Prevention (CDC). Alcohol use and your health 2014 [cited 2015 October 12]. Available from: http://www.cdc.gov/alcohol/fact-sheets/alcohol-use.htm.

[6] Shield KD, Parry C, Rehm J. Chronic Diseases and Conditions Related to Alcohol Use. Alcohol Research. 2014:155–69. 10.35946/arcr.v35.2.06 PMC3908707 24881324

[7] Klatsky AL. Should patients with heart disease drink alcohol? Jama. 2001;285(15):2004–6. Epub 2001/04/20. . 11308439 10.1001/jama.285.15.2004

[8] Koppes LL, Dekker JM, Hendriks HF, Bouter LM, Heine RJ. Moderate alcohol consumption lowers the risk of type 2 diabetes: a meta-analysis of prospective observational studies. Diabetes care. 2005;28(3):719–25. Epub 2005/03/01. . 15735217 10.2337/diacare.28.3.719

[9] Alkerwi A, Boutsen M, Vaillant M, Barre J, Lair ML, Albert A, et al Alcohol consumption and the prevalence of metabolic syndrome: a meta-analysis of observational studies. Atherosclerosis. 2009;204(2):624–35. Epub 2008/12/17. 10.1016/j.atherosclerosis.2008.10.036 . 19084839

[10] Elkind MS, Sciacca R, Boden-Albala B, Rundek T, Paik MC, Sacco RL. Moderate alcohol consumption reduces risk of ischemic stroke: the Northern Manhattan Study. Stroke; a journal of cerebral circulation. 2006;37(1):13–9. Epub 2005/11/25. 10.1161/01.STR.0000195048.86810.5b . 16306464

[11] Sanchez M, De La Rosa M, Blackson TC, Sastre F, Rojas P, Li T, et al Pre- to postimmigration alcohol use trajectories among recent Latino immigrants. Psychology of addictive behaviors: journal of the Society of Psychologists in Addictive Behaviors. 2014;28(4):990–9. Epub 2014/09/23. 10.1037/a0037807 25243834 PMC4274213

[12] Markides KS, Ray LA, Stroup-Benham CA, Trevino F. Acculturation and alcohol consumption in the Mexican American population of the southwestern United States: findings from HHANES 1982–84. American journal of public health. 1990;80 Suppl:42–6. Epub 1990/12/01. 9187581 10.2105/ajph.80.suppl.42 PMC1404517

[13] Center for Disease Control and Prevention (CDC). Alcohol use and binge drinking among women of childbearing age—United States, 2006–2010. MMWR Morb Mortal Wkly Rep. 2012;61(28):534–8. 22810267

[14] Rote SM, Brown RL. Gender differences in alcohol and drug use among Hispanic adults: the influence of family processes and acculturation. Journal of addictive diseases. 2013;32(4):354–64. Epub 2013/12/12. 10.1080/10550887.2013.859452 24325769 PMC3884895

[15] Zemore SE. Acculturation and alcohol among Latino adults in the United States: a comprehensive review. Alcoholism, clinical and experimental research. 2007;31(12):1968–90. Epub 2007/11/24. 10.1111/j.1530-0277.2007.00532.x . 18034692

[16] Sanchez J. Alcohol use among Latino migrant workers in South Florida. Drug and alcohol dependence. 2015;151:241–9. Epub 2015/04/22. 10.1016/j.drugalcdep.2015.03.025 . 25891232

[17] Schwartz SJ, Zamboanga BL, Tomaso CC, Kondo KK, Unger JB, Weisskirch RS, et al Association of acculturation with drinking games among Hispanic college students. The American journal of drug and alcohol abuse. 2014;40(5):359–66. Epub 2014/09/06. 10.3109/00952990.2014.910521 . 25192203

[18] Salas-Wright CP, Clark TT, Vaughn MG, Cordova D. Profiles of acculturation among Hispanics in the United States: links with discrimination and substance use. Social psychiatry and psychiatric epidemiology. 2015;50(1):39–49. Epub 2014/05/06. 10.1007/s00127-014-0889-x 24791924 PMC4286416

[19] Jones L, Bates G, McCoy E, Bellis MA. Relationship between alcohol-attributable disease and socioeconomic status, and the role of alcohol consumption in this relationship: a systematic review and meta-analysis. BMC public health. 2015;15:400 Epub 2015/05/01. 10.1186/s12889-015-1720-7 25928558 PMC4409704

[20] Erskine S, Maheswaran R, Pearson T, Gleeson D. Socioeconomic deprivation, urban-rural location and alcohol-related mortality in England and Wales. BMC public health. 2010;10:99 Epub 2010/02/27. 10.1186/1471-2458-10-99 20184763 PMC2841677

[21] Probst C, Roerecke M, Behrendt S, Rehm J. Gender differences in socioeconomic inequality of alcohol-attributable mortality: A systematic review and meta-analysis. Drug and alcohol review. 2015;34(3):267–77. Epub 2014/08/12. 10.1111/dar.12184 . 25109218

[22] Cook WK, Caetano R. Ethnic drinking cultures, gender, and socioeconomic status in Asian American and Latino drinking. Alcoholism, clinical and experimental research. 2014;38(12):3043–51. Epub 2015/01/13. 10.1111/acer.12573 25581659 PMC4293044

[23] Kochhar R, Cilluffo A. Key findings on the rise in income inequality within America’s racial and ethnic groups2018. Available from: http://www.pewresearch.org/fact-tank/2018/07/12/key-findings-on-the-rise-in-income-inequality-within-americas-racial-and-ethnic-groups/.

[24] Chartier K, Caetano R. Ethnicity and health disparities in alcohol research. Alcohol research & health: the journal of the National Institute on Alcohol Abuse and Alcoholism. 2010;33(1–2):152–60. Epub 2011/01/07. 21209793 PMC3887493

[25] van Oers JA, Bongers IM, van de Goor LA, Garretsen HF. Alcohol consumption, alcohol-related problems, problem drinking, and socioeconomic status. Alcohol Alcohol. 1999;34(1):78–88. Epub 1999/03/13. . 10075406 10.1093/alcalc/34.1.78

[26] Black SA, Markides KS. Acculturation and alcohol consumption in Puerto Rican, Cuban-American, and Mexican-American women in the United States. American journal of public health. 1993;83(6):890–3. Epub 1993/06/01. 8498630 10.2105/ajph.83.6.890 PMC1694747

[27] Sorlie PD, Aviles-Santa LM, Wassertheil-Smoller S, Kaplan RC, Daviglus ML, Giachello AL, et al Design and implementation of the Hispanic Community Health Study/Study of Latinos. Ann Epidemiol. 2010;20(8):629–41. 10.1016/j.annepidem.2010.03.015 20609343 PMC2904957

[28] Lavange LM, Kalsbeek WD, Sorlie PD, Aviles-Santa LM, Kaplan RC, Barnhart J, et al Sample design and cohort selection in the Hispanic Community Health Study/Study of Latinos. Ann Epidemiol. 2010;20(8):642–9. 10.1016/j.annepidem.2010.05.006 20609344 PMC2921622

[29] SAS Institute Inc. SAS® Software Version 9.3. Cary, NC2011.

[30] Caetano R, Ramisetty-Mikler S, Rodriguez LA. The Hispanic Americans Baseline Alcohol Survey (HABLAS): rates and predictors of alcohol abuse and dependence across Hispanic national groups. J Stud Alcohol Drugs. 2008;69(3):441–8. 18432387 10.15288/jsad.2008.69.441 PMC2553043

[31] Glass JE, Rathouz PJ, Gattis M, Joo YS, Nelson JC, Williams EC. Intersections of poverty, race/ethnicity, and sex: alcohol consumption and adverse outcomes in the United States. Social psychiatry and psychiatric epidemiology. 2017;52(5):515–24. Epub 2017/03/30. 10.1007/s00127-017-1362-4 28349171 PMC5862428

[32] Lee CS, Colby SM, Rohsenow DJ, Lopez SR, Hernandez L, Caetano R. Acculturation stress and drinking problems among urban heavy drinking Latinos in the Northeast. J Ethn Subst Abuse. 2013;12(4):308–20. 10.1080/15332640.2013.830942 24215224 PMC3976985

[33] Lopez EB, Yamashita T. The relationship of education and acculturation with vigorous intensity leisure time physical activity by gender in Latinos. Ethnicity & health. 2017:1–16. Epub 2017/03/10. 10.1080/13557858.2017.1294664 PubMed PMID:28277017. 28277017

[34] Collins SE. Associations Between Socioeconomic Factors and Alcohol Outcomes. Alcohol Res. 2016;38(1):83–94. 27159815 10.35946/arcr.v38.1.11 PMC4872618

[35] Mulia N, Ye Y, Zemore SE, Greenfield TK. Social disadvantage, stress, and alcohol use among black, Hispanic, and white Americans: findings from the 2005 U.S. National Alcohol Survey. J Stud Alcohol Drugs. 2008;69(6):824–33. 18925340 10.15288/jsad.2008.69.824 PMC2583375

